# Ingestion of Fluids of the Ocular Surface Containing Eye Drops of Imidazole Derivatives—Alpha Adrenergic Receptor Agonists as Paragons

**DOI:** 10.3390/ph17060758

**Published:** 2024-06-09

**Authors:** Ivan Šoša

**Affiliations:** Department of Anatomy, Faculty of Medicine, University of Rijeka, 51000 Rijeka, Croatia; ivan.sosa@uniri.hr

**Keywords:** alpha-2 adrenergic receptors agonists, eye drops, fluids of the ocular surface, ingestion, ocular surface fluid, tetrahydrozoline

## Abstract

Accidental poisonings by ingesting conjunctival fluid mixed with eye drops commonly involve alpha-2 adrenergic receptor agonists and tetrahydrozoline. These substances are recognized in commonly reported ingestions. Victims of all ages, otherwise in good health, often present as pale and lethargic to the emergency department (ED) after unintentionally ingesting topical eye medication. While eye drop poisoning cases in childhood include accidents during the play and poisonings in adults mean either suicide attempts or side effects caused by the systemic absorption of the substance, fluid of the ocular surface is a risk to all age groups. With this in mind, this study aimed to summarize data in the literature on tetrahydrozoline and alpha-2 adrenergic receptor agonists as dangerous medications, even when administered in low-bioavailability forms, such as eye drops. With this aim, a Preferred Reporting Items for Systematic Reviews and Meta-Analyses (PRISMA)-compliant systematic review of relevant studies was conducted. A search of PubMed, Scopus, Web of Science, and EBSCOhost yielded nine studies that met the rigorous inclusion criteria. The primary studies were subject to a meta-analysis once a quality appraisal of the studies and a narrative synthesis of the extracted data had been conducted. The author hopes that this information will provide observations that will lead to better designs for over-the-counter eye drops, off-label drug usage policies, and parental attention.

## 1. Introduction

The pharmaceutical effects of imidazole derivatives have been exploited for decades. Simultaneously with the discovery of clonidine, research on imidazoline receptors brought about some knowledge on the systemic biodistribution and oral bioavailability of imidazole derivatives [1,2]. Furthermore, knowledge about imidazoline receptors has invigorated the idea that drugs such as clonidine and tetrahydrozoline have much toxicity due to their binding to imidazoline receptors [2,3,4,5]. Clonidine, as a prototypical central alpha-2 adrenergic agonist, functions through its agonism, while tetrahydrozoline is a selective agonist of alpha-1 adrenergic receptors [6]. These agents stimulate adrenergic receptors, with a slight difference in their pharmacotherapeutic features. 

Even a microinjection of clonidine in the nucleus tractus solitarii (NTS) and locus coeruleus produces a decrease in blood pressure but a series of unfavorable side effects as well (e.g., sedation, dry mouth) [3,4,7]. Clonidine is widely used in pharmacology to treat high blood pressure, attention-deficit/hyperactivity disorder (ADHD), substance withdrawal (alcohol, opioids, or nicotine), menopausal flushing, diarrhea, spasticity, and certain pain conditions [8,9,10]. As a boost to the action of alpha-2 adrenergic agonists, clonidine significantly decreases intraocular pressure (IOP), which is highly relevant.

Tetrahydrozoline, on the other hand, causes vasoconstriction via alpha-1 adrenergic receptors and relieves ocular and nasal congestion [6]. All of this explains the role of these drugs in adult and pediatric medicine.

## 2. Eye Drops as Fluids of the Ocular Surface

For the perusal of this review, fluids of the ocular surface (OS) are given as in an article by Cher Franzco (2012) [11], where the OS is conceptually a compartment. Elsewhere in the literature, this is referred to as “tear film”.

- The palpebral fissure is layered with an ever-present lipid sealant, which isolates a much-aqueous pool (MAP) from the atmosphere, preventing its evaporation mitigation. The rate of tear secretion is controlled by parasympathetic and sympathetic innervation [12].

**Figure 1:**
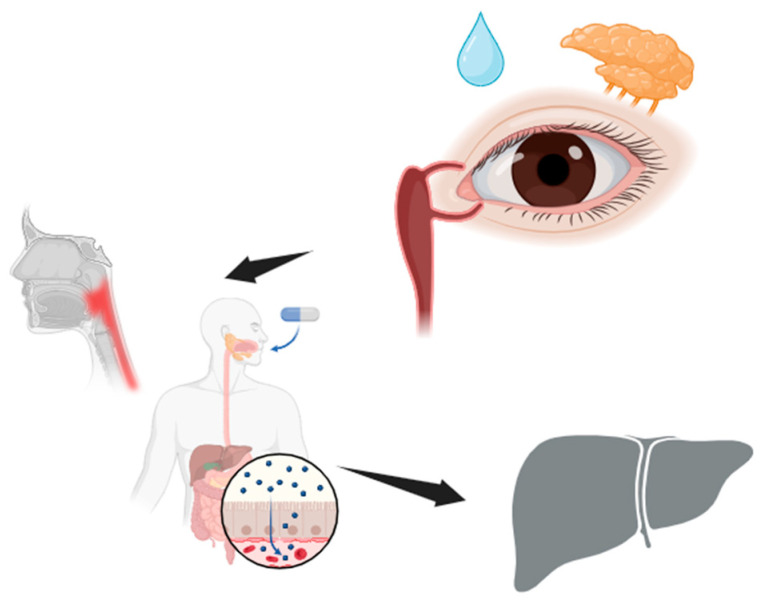
Path of an eye drop via the nasolacrimal duct to the liver. (Created with BioRender.com, accessed on 13 May 2024).

In the above concept of lipid film and MAP, tears are an essential component of the precorneal tear film. The mixture of tears and eye drops drains through a small communication canal between the maxillary and lacrimal bones. Through this communication, the nasolacrimal duct (tear duct) content from the OS is drained through tears flowing from the lacrimal sac of the eye into the nasal cavity (Figure 1). Blinking and tear flow can reduce this even more, so that less than five percent of conventional eye drops finally reach the systemic circulation [13]. Therefore, various strategies have been adopted to improve ocular drug bioavailability and reduce systemic side effects [14,15,16,17].

There, it is rapidly absorbed by the nasal mucosa [11,18,19]. Up to 80% of the applied drug(s) may diffuse into the systemic circulation by crossing the highly vascularized nasopharyngeal mucosa with respect to corneal and conjunctival absorption [20]. The remaining, unabsorbed portion of this fluid ultimately ends up in the throat for swallowing. In that case, it reaches the liver, which is responsible for the selective uptake, concentration, metabolism, and excretion of the majority of drugs. In the context of the toxicity of ophthalmic preparations containing imidazole derivatives with alpha-adrenergic activity, all clinical consequences reflect the extent and rate at which medications in the ocular surface fluid are absorbed and become available at the endpoint—bioavailability. This feature is even more significant considering that alpha-2 adrenergic receptor agonists and tetrahydrozoline are recognized as commonly reported ingestions [2,4,5,21,22]. The bioavailability of orally taken drugs is more important than that of those administered as eye drops and less important than that of those taken intravenously [23,24]. However, the route of administration affects bioavailability, so when a medication is administered intravenously, its bioavailability is 100%. When routes other than intravenous ones are used, its bioavailability is lower due to intestinal epithelium absorption and first-pass metabolism in the liver. In cases where alpha-adrenergic agonists are administered systemically, no liver enzyme induction is recorded in humans. For comparison, an exact imidazole derivative induced liver enzymes in experimental animals [2,7,25,26]. However, the oral administration of high doses induced vomiting in some mammals [27]. In general, ocular medications are absorbed via the ocular or nasal mucous membranes and do not undergo first-pass metabolism in the liver [28]. Of course, a recently adopted concept of the liver–eyes connection could shed a whole new light on this system [29,30]. It should also be said that the “first-pass” principle is a general pharmacokinetic principle that signifies that a medication undergoes metabolism at a specific location in the body [31].

In this regard, one German study assessed the absorption of other imidazolines in animals and adult humans and found the elimination (65% urine and 22% feces) was “finished” at 96 h post-ingestion of a 390 mcg dose [32]. The data in this review were collected separately for clonidine and tetrahydrozoline [33,34,35]. 

## 3. Eye Drops—Illicit Use and Abuse

If not mitigated, the systemic effects of imidazole derivative-containing eye drops should be regarded as consequences of intentional ingestion, not accidental ingestion of the conjunctival fluid [11]. Even though sympatholytic effects such as CNS depression, respiratory depression, hypotension, bradycardia, miosis, hypothermia, and hyporeflexia have been well described in the pediatric literature [36], signs of central nervous stimulation with an elevation in muscle tone and tonic–clonic spasms accompanied by death are also described [37,38]. A case of pediatric peroral tetrahydrozoline poisoning even manifested with hypoglycemia and hypothermia. This was attributed to poor oral intake and a blunted central sympathetic response to cold and hypoglycemia [39]. This brings to the forefront the paradigm that drugs intended for local application should not be administered systemically and the other way around [2,40,41,42].

### Poisoning by Ingestion of Eye Drops

**Figure 2:**
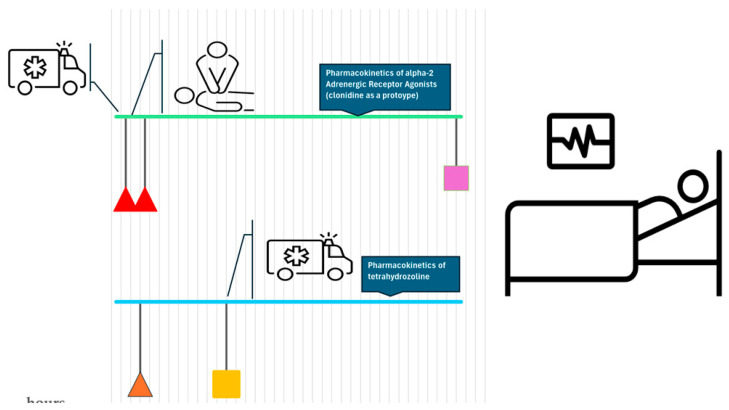
Time course of events relevant in cases of poisoning with low-bioavailable imidazole derivatives. -⧌ (a triangular-shaped mark) indicates the half-life of the xenobiotic in serum (3–7 h for clonidine and 6 h for tetrahydrozoline); □ (a rectangle-shaped mark) represents the last sample with a detectable amount of xenobiotic (by GC/MS) (72 h for clonidine and 24 h for tetrahydrozoline) [33,34,35]. Symptoms of clonidine poisoning typically occur in two phases. Firstly, somnolence, difficulties breathing, hypotension, bradycardia, and miosis manifest. Alterations in the level of consciousness usually are present within the first one and a half hours. In the second phase, any cardiovascular manifestations usually occur within 4 h [5,38]. Symptoms of tetrahydrozoline overdose may occur and persist for up to 72 h (fast or slow heartbeat; headache; irritability; low body temperature; nausea/ vomiting; nervousness, tremors; seizures; weakness) [52]. In either case, medical monitoring/ hospitalization may be optional.

Upon ingestion of small amounts of tetrahydrozoline, toddlers may experience lethargy and difficulty breathing. More significant amounts may result in similar outcomes in teenagers and adults. In young children, unintentional ingestion of both alpha-2 receptor agonists and tetrahydrozoline eye drops is common. However, exposure to the letter has been rising in the adult population. While poisoning symptoms are uncommon after applying these drops to the eyes, these agents can produce significant poisoning if taken orally. Moreover, alpha-agonist toxicity is similar. These substances can be associated with CNS depression, bradycardia, and hypotension, but with proper medical care, patients usually do very well [43] (Figure 2). Alpha-agonists’ toxicity may occur after ingestion of pills [44,45] or through the skin patches [5,46]. Be that as it may, this review focuses on the ingestion of conjunctival fluid mixed with eye drops. This may occur due to accidental ingestion (in the pediatric population) or intentionally (as overdoses in suicides or other drug abuse) [46,47,48]. In a recent review of tetrahydrozoline poisoning cases by Menshawey and Menshawey [35], their findings were the same. Though, unlike selective alpha-2 agonists (usually prescription medications) [22], tetrahydrozoline drops are easily accessible and do not call for pharmacists or police investigators. For those reasons, this substance is likely to be used as an agent to facilitate homicide or suicide.

Conversely, both drugs may trigger or aggravate abnormal adverse effects classically associated with alpha-2 adrenergic and imidazoline receptor agonists [21,28]. It is established, however, that even when imidazole derivatives are used as a nasal decongestant in children (with doses prescribed to adults), these medications can cause systemic toxic effects such as lethargy, bradycardia, miosis, hypotension, and respiratory depression [25,49,50]. In brief, the harmful effects of all imidazolines include central nervous system (CNS) depression [51]. Poisoned victims can develop sleepiness and difficulty breathing. When more significant amounts are ingested, heart rhythm abnormalities can be registered, and life-threatening breathing problems are possible [52,53]. Patients poisoned with a therapeutic ophthalmic application of alpha-2 agonists (such as clonidine) manifest with bradycardia, altered mental status, and apnea. The most common demonstration of the toxic effects of alpha-2 adrenergic receptor agonists is much like that of tetrahydrozoline [54]. Emergency department (ED) professionals should be trained to evaluate possible eye drop poisoning [52,55].

The manifestations of poisoning depend on the nature of the poison, the amount taken, and the properties (like age, development, and underlying health) of the person who takes it. So, this study aims to summarize literature reports on tetrahydrozoline and alpha-2 adrenergic receptor agonists (clonidine taken as a prototypical medication) as harmful drugs, even in the form of fluid of the OS tainted with eye drops containing imidazole derivatives with the activity of alpha-adrenergic receptor agonists.

## 4. Results

After identifying the search terms, before screening, 217 studies were removed either as part of the deduplication process (*n* = 136), due to them being secondary publications based on the title (*n* = 65), or because they are not written in English (*n* = 16). Some studies identified that reported cases in a non-English language were excluded from this systematic review at the beginning of the screening process [5,38,56]. One study was written declaratively in English, but the quality of the language was too low [57]. One case of brimonidine poisoning that presented as respiratory depression was excluded from this review as it was an incomplete study.

At the end of the study selection process, 2 out of 201 full-text studies matched the inclusion criteria. Both were completely original research papers.

### 4.1. Study Characteristics

From the search, there were only two original studies that matched the inclusion criteria for this review [58,59], and only one of them directly focused on clonidine [58].

A study by Sihota et al. [58] tested the efficacy and safety of the drug combination pilocarpine 1% with clonidine 0.06% or clonidine 0.125% compared to that of timolol 0.25% in a randomized, long-term study. Sixty primary open-angle glaucoma patients were included and randomly assigned to three parallel study groups. All the clonidine-treated patients experienced a reduction in IOP, but there was no statistically significant difference in pulse rate, blood pressure, and visual acuity changes between the study groups.

Due to brimonidine’s similarity to clonidine, a study that focused on brimonidine tartrate poisoning in children was also included in this review. It met the criteria for inclusion by having “clonidine” in the abstract. There were 753 participants included in this study by Lai Backer et al. [59], of which 200 reports involved children ≤5 years of age. There were no reported deaths; however, signs of CNS depression were reported (Table 1).

The extremely rigorous “inclusion criteria” were met by only 2 out of 201 full texts (0.99%). For that reason, the conclusions based on those data can hardly convince the readers of the real peril of the adverse effects of an OS fluid “enriched” in alpha-2 agonists. However, the initial search of the literature (performed by the search engines) retrieved numerous publications. Information on the included studies, the characteristics of the populations included, and the primary outcomes and interventions are brought together in Table 2.

In the process of retrieving tetrahydrozoline-related original research papers, the search engines retrieved two original studies as well. These considered the use of tetrahydrozoline-containing eyedrops to falsify positive urine drug tests [60,61]. The exact topic was not related to “ocular surface fluid”, so both of these studies were excluded.

### 4.2. Risk of Bias

A fixed set of five different domains of bias was used to assess various aspects of the study design.

**Figure 3:**
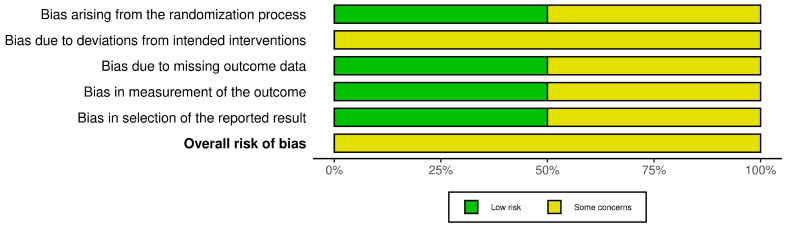
Summary of the risk of bias for the studies included in the meta-analysis identified by the author.

A series of questions (‘signaling questions’) were considered in each domain with the aim of eliciting information about different aspects of the design, conduct, and reporting of the relevant study. Judgments about the risk of bias arising from each domain were based on answers to the signaling questions. Judgments can be a “low” or ‘“high” risk of bias or can express “some concerns”. No domain in the two studies in this review was judged to present a “high” risk of bias. Some concerns were related to the “performance bias” for both included studies, as both express a deviation from the expected intervention (in both cases, there were poisoning and adverse outcomes). A survey by Sihota et al. [58] expressed a “selection bias” and a “detection bias” on account of sampling and grouping/sorting the participants. Lai Becker et al. [59] considered only the pediatric population with a fairly blurred methodology and doubt regarding outcomes. Summary of “risk of bias” assessments is presented in Figure 3. 

## 5. Discussion

The results of this literature review yield a negligible number of only two out of 201 full texts (<1%) dealing with the ingestion of an ocular surface fluid. Nevertheless, data from these studies were extracted and included in this meta-analysis, indicating that problems arising from the use of imidazole derivatives with the activity of alpha-adrenergic agonists are rarely reported.

This could be because today’s understanding of frequently used imidazole derivatives refers to those used almost exclusively for local therapy, with the exception of some alpha-adrenergic agonists used as pills or patches [44,45,46].

Signs and symptoms identified in case reports of poisonings include drowsiness, comas, hyperactivity, hypertension, hypotension, bradycardia, respiratory depression, and apnea. The studies included in this review (one of them) reported drowsiness, ataxia, irritability, bradycardia, and hypotension. While case reports specify the onset of symptoms within minutes to hours of ingestion, none of the two included studies does so for the ingestion of ocular surface fluid.

The infrequent relation of these substances to death is the result of the relatively low toxicity associated with their topical administration. Numerous case reports have been issued documenting the dangers of ingesting topical over-the-counter products, including alpha-agonist eye drops. These products are capable of depressing the CNS due to the preferential effects of the drugs on alpha-receptors, with an effect similar to that of clonidine.

### 5.1. Case Reports of Misuse

In the scope of this paper, the terms “misuse” and “abuse” were used, as in the paper of Hughes et al. [62], as it deals with ingestion and highlights “unforeseen use or use not as intended” of certain substances. On the other hand, the term “abuse” was defined as an action that intentionally causes harm or injury through the improper use of a substance.

Case report studies screened as full texts during the systematic review of the literature revealed that eye drops can be a severe health hazard to children, even though some cases arise in adults (intentional use) [52,63]. The context of a health hazard for children is even more relevant in the study of tetrahydrozoline as it is available over the counter. Brimonidine and apraclonidine, drugs similar to clonidine, are prescribed as eye drops for the treatment of glaucoma. These drugs can cause severe poisoning within 30 min [8,59,64]. Be that as it may, most of the cases identified during the systematic search of the literature were accidents that occurred during play. Frequently, eye drops are not packaged in child-resistant containers. Moreover, clonidine is used for a number of conditions. The literature also suggests that patients with signs of CNS depression should be regarded as victims of poisoning with imidazole compounds [56,57,58,59].

### 5.2. Case Reports of Abuse

The medications considered here(tetrahydrozoline, in particular) are used to facilitate sexual crimes as Drug-Facilitated Sexual Assault (DFSA) agents. [65,66,67]. Tetrahydrozoline is not used as a DFSA agent for its ability to hinder the victim but because of its ability to produce a false negative urine drug test for benzodiazepines [60,61,68]. Building on that, ophthalmic preparations containing imidazole derivatives with alpha-adrenergic activity have received widespread media coverage when eye drops and nasal decongestant sprays containing them became involved in some significant crime prosecutions [48,69,70].

The literature also recognizes the use of these medications as DFSA agents. The latter term refers to any content ingestion of psychoactive substances for criminal reasons of an economic or sexual nature [67]. In the context of using eye drops in relation to any crime, bear in mind that good penetration will ensure active concentrations at the expense of absorption and removal by the vascular system [4,5,71,72,73].

Reports of the recreational abuse of eye drops containing tropicamide or to alter positive urine drug tests suggest the ingenuity of the “criminal mind” [74,75,76].

These studies were not included in this review as they did not meet the criteria for inclusion. However, their common finding was that tetrahydrozoline-containing eye drops alter drug tests for urine samples containing some drugs when poured into these samples [60,61]. The same is true for cases where the patient stated that they used Super Glue™ by mistake instead of eye drops, but this was not considered in this review [77]. This mistake is always possible in circumstances where the off-label usage of medications (including eye drops) can be as high as 90% in the pediatric population and 40% in adults (estimation) [78,79]. Epidemiological studies on eye drop accidents do not cover accidental (or intentional) swallowing of an ocular surface fluid. Hence, the above knowledge might seem outside of this study’s scope.

### 5.3. Limitations

However, this indicates the first possible limitation of this meta-analysis. The inclusion criteria might be too harsh, so the review may look as though it has been poorly or superficially executed. This apparent failure to consider critical studies was hopefully avoided by considering case reports of accidental poisonings and intentional overdoses in more comprehensive databases. This cannot convince readers to be concerned about the adverse effects of the regular use of these drugs. Of course, there is always a chance of bias on the part of the meta-analyst, and overstatements of the strength and precision of the results can all contribute to invalid meta-analyses [80].

## 6. Materials and Methods

The Preferred Reporting Items for Systematic Reviews and Meta-Analyses (PRISMA) guidelines were considered for conducting the systematic review for this article. The data were gathered from databases that are available via institutional access to the University of Rijeka. The articles indexed in PubMed, Web of Science—core collection, Scopus, and EBSCOhost were included from their inception until 21 February 2024. The search strategy included journal articles and conference proceedings written in English. The title/abstract/keywords of the studies were searched using the terms presented in Table 3. The Boolean operator AND was used while searching.

### 6.1. Inclusion Criteria

- Articles indexed in PubMed, Web of Science—core collection, Scopus, and EBSCOhost.
- Articles published since the inception of the respective database until 7 May 2024.
- The papers must leverage technologies like artificial intelligence (AI) and machine learning (ML) and must focus on ingested lacrimal liquid.
- The studies must be related to drugs for ocular use or for use in the form of eye drops.
- The articles must be accessible as full texts.
- Inclusion in the meta-analysis required studies to be related to eye drops.

### 6.2. Exclusion Criteria

- Articles containing partial texts and articles available in languages other than English.
- Secondary (desk) research.
- Articles that do not focus on tetrahydrozoline or alpha-adrenergic receptor agonists.

Applying the criteria mentioned above further decreased the number of papers available for this study.

### 6.3. Literature Review and Data Extraction

**Figure 4:**
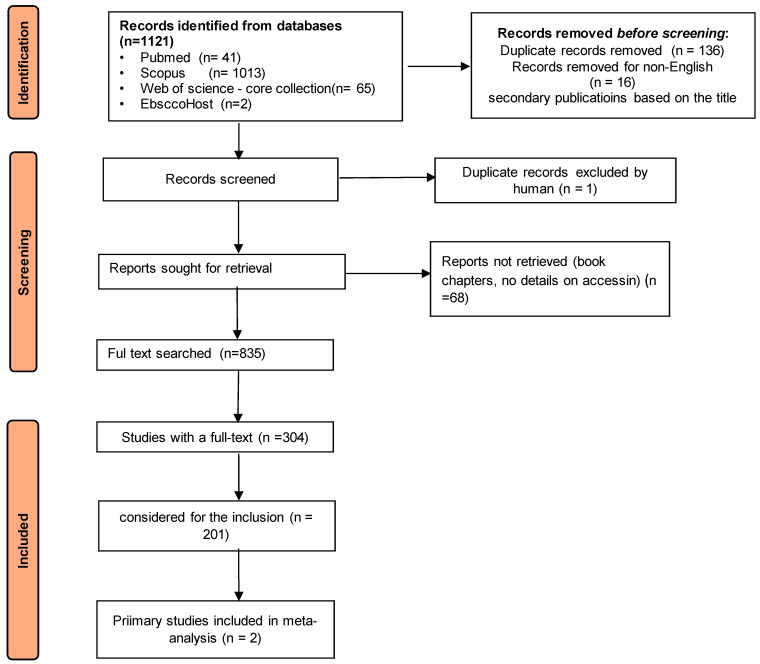
Diagram of Preferred Reporting Items for Systematic Reviews and Meta-Analyses (PRISMA)-compliant systematic review used in this study.

The papers were meticulously weighed, and for the qualitative extraction, a PRISMA flow chart (Figure 4) was used. The collected data were abstracted into actionable resources.

The broad inclusion criteria were narrowed down as the literature search advanced. In addition, the quality and risk of bias of the studies included in the meta-analysis were assessed using commonly used critical appraisal tools (RoB 2: A revised Cochrane risk-of-bias tool for randomized trials was used in this case).

## 7. Conclusions

Ingestions of ocular surface fluid containing ophthalmic preparations of imidazole derivatives with alpha-adrenergic activity seen in adults or pediatric patients produce clinical effects similar to those seen with clonidine poisonings. For this reason, symptoms should be treated precisely like they would be for patients with clonidine intoxications.

The information shared in the review regarding imidazole derivatives with alpha-adrenergic activity is helpful and informative for healthcare providers and, in the end, beneficial to patients who regularly use these drugs for relieving eye disorders [81]. Medical students could also benefit from this review. The findings of this literature review should be informative for the civil sector and government authorities dealing with illicit drugs.

## 8. Future Directions

Some specific public health measures (e.g., education of parents, improving strategies for eye drop administration) should be designed according to the findings of this review.

Both tetrahydrozoline and alpha-2 agonists are infrequent findings in toxicology testing but have been reported in multiple crimes. This highlights the importance of including these medications in comprehensive post-mortem toxicology testing.

## Figures and Tables

**Table 1:** Symptoms/signs of alpha-adrenergic agonist toxicity.

Toxicity Signs and Symptoms	Sihota et al. [58]	Lai Becker et al. [59]
local symptoms	10%	
symptoms of systemic toxicity	0%	58%
unrelated to exposure		3.4%
drowsiness		41%
ataxia		4.5%
irritability		4%
bradycardia		4%
hypotension		4%

**Table 2:** Characteristics of the original research studies that met the inclusion criteria.

Included Study	Population and Methodology		Agent	Interventions	Outcomes
Sihota et al. [58]	*n* = 60; miscellanies	prospective, randomized study	drug combination pilocarpine 1% with clonidine 0.06% or clonidine 0.125% versus timolol 0.25%	efficacy and safety of the drug combination	Signs and symptoms of the depression of central nervous system
Lai Becker et al. [59]	*n* = 753; pediatric (185 ≤5 years)	case series + FDA database	brimonidine	brimonidine tartrate poisoning in children	drowsiness, ataxia, pallor, irritability, hypotension, bradycardia, miosis, and respiratory depression.

**Table 3:** Search terms used in the screening of databases.

Search Term #1	Operator	Search Term #2
Tetrahydrozoline	AND	homicide
Tetrahydrozoline	AND	suicide
Tetrahydrozoline	AND	Accidental poisoning
Tetrahydrozoline	AND	accident
Tetrahydrozoline	AND	autopsy
Clonidine	AND	homicide
Clonidine	AND	suicide
Clonidine	AND	Accidental poisoning
Clonidine	AND	accident
Clonidine	AND	autopsy

## Data Availability

Available on request.

## References

[1] Bousquet P., Hudson A., Garcia-Sevilla J.A., Li J.X. (2020). Imidazoline Receptor System: The Past, the Present, and the Future. Pharmacol. Rev..

[2] Gujjarappa R., Kabi A.K., Sravani S., Garg A., Vodnala N., Tyagi U., Kaldhi D., Velayutham R., Singh V., Gupta S., Swain B.P. (2022). Overview on Biological Activities of Imidazole Derivatives. Nanostructured Biomaterials.

[3] Lowry J.A., Brown J.T. (2014). Significance of the imidazoline receptors in toxicology. Clin. Toxicol..

[4] Norman K., Nappe T.M. (2024). Alpha Receptor Agonist Toxicity. StatPearls.

[5] Manzon L., Nappe T.M., DelMaestro C., Maguire N.J. (2017). Clonidine toxicity. StatPearls.

[6] Hosten L.O., Snyder C. (2020). Over-the-Counter Ocular Decongestants in the United States—Mechanisms of Action and Clinical Utility for Management of Ocular Redness. Clin. Optom..

[7] Farkouh A., Frigo P., Czejka M. (2016). Systemic side effects of eye drops: A pharmacokinetic perspective. Clin. Ophthalmol..

[8] Mandelbaum D.E., Swaiman K.F., Ashwal S., Ferriero D.M., Schor N.F., Finkel R.S., Gropman A.L., Pearl P.L., Shevell M.I. (2017). Attention Deficit–Hyperactivity Disorder. Swaiman’s Pediatric Neurology.

[9] Eadie R., McKenzie C.A., Hadfield D., Kalk N.J., Bolesta S., Dempster M., McAuley D.F., Blackwood B., on behalf of UK ALERT-ICU study investigators (2023). Opioid, sedative, preadmission medication and iatrogenic withdrawal risk in UK adult critically ill patients: A point prevalence study. Int. J. Clin. Pharm..

[10] Hermenau M., Stamper B., Leung K., Pomm R., Guerrier C., Cammilleri J., Johnson B. (2023). A Retrospective Comparison of the Effectiveness of Buprenorphine Versus Baclofen for Acute Opioid Withdrawal. HCA Healthc. J. Med..

[11] Cher I. (2012). Fluids of the ocular surface: Concepts, functions and physics. Clin. Exp. Ophthalmol..

[12] Masoudi S. (2022). Biochemistry of human tear film: A review. Exp. Eye Res..

[13] Ahmed S., Amin M.M., Sayed S. (2023). Ocular Drug Delivery: A Comprehensive Review. AAPS PharmSciTech.

[14] Gugleva V., Andonova V. (2023). Recent Progress of Solid Lipid Nanoparticles and Nanostructured Lipid Carriers as Ocular Drug Delivery Platforms. Pharmaceuticals.

[15] Wang J.J., Liu X.X., Zhu C.C., Wang T.Z., Wang S.Y., Liu Y., Pan X.Y., Liu M.H., Chen D., Li L.L. (2023). Improving ocular bioavailability of hydrophilic drugs through dynamic covalent complexation. J. Control Release.

[16] Aschenbrenner D.S. (2024). Preventing Wrong Route Errors: Eye and Ear Drops. AJN Am. J. Nurs..

[17] Muller L., Jensen B.P., Bachmann L.M., Wong D., Wells A.P. (2020). New technique to reduce systemic side effects of timolol eye drops: The tissue press method-Cross-over clinical trial. Clin. Exp. Ophthalmol..

[18] Agarwal P., Rupenthal I.D. (2023). Non-aqueous formulations in topical ocular drug delivery—A paradigm shift?. Adv. Drug Deliv. Rev..

[19] Zhang J.H., Su Y.J., Wu J., Wang H.D. (2024). Recent advances in ocular lubrication. Friction.

[20] Ramsay E., Del Amo E.M., Toropainen E., Tengvall-Unadike U., Ranta V.P., Urtti A., Ruponen M. (2018). Corneal and conjunctival drug permeability: Systematic comparison and pharmacokinetic impact in the eye. Eur. J. Pharm. Sci..

[21] Ávila Reyes D., García Pasichana B.D., Galvis Mejía J.C., Gómez González J.F., Villadiego M., Aguirre Flórez M., González Cuellar J.A. (2021). Toxicological diagnosis in the critical patient: The challenge. Rev. Médica Risaralda.

[22] Giovannitti J.A., Thoms S.M., Crawford J.J. (2015). Alpha-2 adrenergic receptor agonists: A review of current clinical applications. Anesth. Prog..

[23] Lanier O.L., Manfre M.G., Bailey C., Liu Z., Sparks Z., Kulkarni S., Chauhan A. (2021). Review of Approaches for Increasing Ophthalmic Bioavailability for Eye Drop Formulations. AAPS PharmSciTech.

[24] Law S.K., Lee D.A. (2020). Ocular pharmacology. Glaucoma Medical Therapy-Principles and Managemen.

[25] Teli P., Sahiba N., Sethiya A., Soni J., Agarwal S. (2022). Imidazole derivatives: Impact and prospects in antiviral drug discovery. Imidazole-Based Drug Discov..

[26] Rakhshan A., Rahmati Kamel B., Saffaei A., Tavakoli-Ardakani M. (2023). Hepatotoxicity Induced by Azole Antifungal Agents: A Review Study. Iran. J. Pharm. Res..

[27] Adeyemi O.S., Molefe-Nyembe N.I., Eseola A.O., Plass W., Shittu O.K., Yunusa I.O., Atolani O., Evbuomwan I.O., Awakan O.J., Suganuma K. (2021). New Series of Imidazoles Showed Promising Growth Inhibitory and Curative Potential Against Trypanosoma Infection. Yale J. Biol. Med..

[28] Vaajanen A., Vapaatalo H. (2017). A Single Drop in the Eye—Effects on the Whole Body?. Open Ophthalmol. J..

[29] Yuan T.H., Yue Z.S., Zhang G.H., Wang L., Dou G.R. (2021). Beyond the Liver: Liver-Eye Communication in Clinical and Experimental Aspects. Front. Mol. Biosci..

[30] Wu J., Duan C., Yang Y., Wang Z., Tan C., Han C., Hou X. (2023). Insights into the liver-eyes connections, from epidemiological, mechanical studies to clinical translation. J. Transl. Med..

[31] Herman T.F., Santos C. (2024). First-Pass Effect. StatPearls.

[32] Rehbinder D., Deckers W. (1969). [Studies on the pharmacokinetics and on the metabolism of 2(2,6-dichlorphenylamino)-2-imidazoline-hydrochloride (St 155)]. Arzneim. -Forsch..

[33] Luo W., Pan J., Chen B., Ma M. (2022). Rapid Determination of Clonidine in Pharmaceutical Preparations by Paper Spray Tandem Mass Spectrometry (PS-MS/MS). Anal. Lett..

[34] Peat J., Garg U. (2010). Determination of tetrahydrozoline in urine and blood using gas chromatography-mass spectrometry (GC-MS). Methods Mol. Biol..

[35] Menshawey E., Menshawey R. (2023). More than meets the eye: A scoping review on the non-medical uses of THZ eye drops. Forensic Sci. Med. Pathol..

[36] Gussow L. (2020). Toxicology Rounds: Unexplained Bradycardia? Consider Imidazolines. Emerg. Med. News.

[37] Johnson M.L., Visser E.J., Goucke C.R. (2011). Massive Clonidine Overdose During Refill of an Implanted Drug Delivery Device for Intrathecal Analgesia: A Review of Inadvertent Soft-Tissue Injection During Implantable Drug Delivery Device Refills and Its Management. Pain Med..

[38] Manzon L., Nappe T.M., DelMaestro C., Maguire N.J. (2024). Clonidine Toxicity. 2023 June 26. StatPearls [Internet].

[39] Tobias J.D. (1996). Central nervous system depression following accidental ingestion of Visine eye drops. Clin. Pediatr..

[40] LiverTox L. Clinical and Research Information on Drug-Induced Liver Injury [Internet]. Bethesda. MD: National Institute of Diabetes and Digestive and Kidney Diseases 2012. https://pubmed.ncbi.nlm.nih.gov/31643176/.

[41] Kartasheva-Ebertz D., Gaston J., Lair-Mehiri L., Massault P.P., Scatton O., Vaillant J.C., Morozov V.A., Pol S., Lagaye S. (2021). Adult human liver slice cultures: Modelling of liver fibrosis and evaluation of new anti-fibrotic drugs. World J. Hepatol..

[42] Zhang Y.B., Xu D., Bai L., Zhou Y.M., Zhang H., Cui Y.L. (2022). A Review of Non-Invasive Drug Delivery through Respiratory Routes. Pharmaceutics.

[43] (2010). Abstracts of the 2010 International Congress of the European Association of Poisons Centres and Clinical Toxicologists, 11–14 May 2010, Bordeaux, France. Clin. Toxicol..

[44] Cibickova L., Caran T., Dobias M., Ondra P., Vorisek V., Cibicek N. (2015). Multi-drug intoxication fatality involving atorvastatin: A case report. Forensic Sci. Int..

[45] Tenenbein M. (2023). The One Pill Can Kill Myth. Pediatr. Emerg. Care.

[46] Broderick-Cantwell J.J. (1999). Case study: Accidental clonidine patch overdose in attention-deficit/hyperactivity disorder patients. J. Am. Acad. Child. Adolesc. Psychiatry.

[47] Bhullar J., Patel A., Chitithoti J., Venter F., Win T., Joolhar F. (2022). Clonidine Overdose as an Unusual Cause of Heart Failure. J. Investig. Med. High Impact Case Rep..

[48] Killian C.A., Roberge R.J., Krenzelok E.P., Stonage C.L. (1997). “Cloniderm” toxicity: Another manifestation of clonidine overdose. Pediatr. Emerg. Care.

[49] van Groen B.D., Krekels E.H.J., Mooij M.G., van Duijn E., Vaes W.H.J., Windhorst A.D., van Rosmalen J., Hartman S.J.F., Hendrikse N.H., Koch B.C.P. (2021). The Oral Bioavailability and Metabolism of Midazolam in Stable Critically Ill Children: A Pharmacokinetic Microtracing Study. Clin. Pharmacol. Ther..

[50] Sharma H.P., Vijayakumar A.R., Velpandian T., Velpandian T. (2016). Systemic Toxicity of Drugs Applied to the Eye. Pharmacology of Ocular Therapeutics.

[51] Commission., Consumer Product Safety (2012). Products Containing Imidazolines Equivalent to 0.08 Milligrams or More. The Federal Register.

[52] Afify O., Suleiman A.M., Mohamed H.G., Saaed O. (2021). Complete atrioventricular block due to ingestion of Visine eye drops. BMJ Case Rep..

[53] Kacinko S., Lamb M. (2022). Tetrahydrozoline: Death by Eyedrops. Toxicol. Anal. Clin..

[54] Amna S., Ohlenschlaeger T., Saedder E.A., Sigaard J.V., Bergmann T.K. (2024). Review of clinical pharmacokinetics and pharmacodynamics of clonidine as an adjunct to opioids in palliative care. Basic. Clin. Pharmacol. Toxicol..

[55] McCord E., Van Hale C., Tang Y.-L., Kaye A.D., Urman R.D., Cornett E.M., Edinoff A.N. (2023). Medical treatments for opioid use disorder. Substance Use and Addiction Research.

[56] Rangan C., Everson G., Cantrell F.L. (2008). Central alpha-2 adrenergic eye drops: Case series of 3 pediatric systemic poisonings. Pediatr. Emerg. Care.

[57] Gunes A., Balik H., Yel S., Kocamaz H., Bosnak M. (2016). Respiratuvar depression after accidental nasal ingestion of brimonidine eye drops in infant. Turk. J. Emerg. Med..

[58] Sihota R., Rajashekhar Y.L., Venkatesh P., Agarwal H. (2002). A prospective, long-term, randomized study of the efficacy and safety of the drug combination pilocarpine 1% with clonidine 0.06% or clonidine 0.125% versus timolol 0.25%. J. Ocul. Pharmacol. Ther..

[59] Lai Becker M., Huntington N., Woolf A.D. (2009). Brimonidine tartrate poisoning in children: Frequency, trends, and use of naloxone as an antidote. Pediatrics.

[60] Pearson S.D., Ash K.O., Urry F.M. (1989). Mechanism of false-negative urine cannabinoid immunoassay screens by Visine eyedrops. Clin. Chem..

[61] Mikkelsen S.L., Ash K.O. (1988). Adulterants causing false negatives in illicit drug testing. Clin. Chem..

[62] Hughes A.R., Grusing S., Lin A., Hendrickson R.G., Sheridan D.C., Marshall R., Zane Horowitz B. (2023). Trends in intentional abuse and misuse ingestions in school-aged children and adolescents reported to US poison centers from 2000–2020. Clin. Toxicol..

[63] Al-Abri S.A., Yang H.S., Olson K.R. (2014). Unintentional pediatric ophthalmic tetrahydrozoline ingestion: Case files of the medical toxicology fellowship at the University of California, San Francisco. J. Med. Toxicol..

[64] Ghaffari Z., Zakariaei Z., Ghazaeian M., Jafari R., Ezoddin N., Yousefi Nouraee H., Navaeifar M.R. (2021). Adverse effects of brimonidine eye drop in children: A case series. J. Clin. Pharm. Ther..

[65] Lusthof K.J., Lameijer W., Zweipfenning P.G. (2000). Use of clonidine for chemical submission. J. Toxicol. Clin. Toxicol..

[66] Stillwell M.E., Saady J.J. (2012). Use of tetrahydrozoline for chemical submission. Forensic Sci. Int..

[67] Moran G.A.C., Ccoscco C.A.C., Alcántara K.J.G., Ramos V.T., Pérez V.C. (2022). Chemical submission in cases of alleged crimes against sexual freedom 2016–2018, Lima, Peru. Span. J. Leg. Med..

[68] Edwards C.N., Tilley D.S., Ayala F. (2023). Drug-Facilitated Sexual Assault With Tetrahydrozoline (Visine): A Case Report. J. Forensic Nurs..

[69] News A. Wisconsin Woman Arrested, Accused of Murdering Friend with Eye Drops. https://abcnews.go.com/amp/US/wisconsin-woman-arrested-accused-murdering-friend-eye-drops/story?id=78148517.

[70] Gitter M.F., Cox R. (2000). Clonidine toxicity in an adolescent patient. J. Miss. State Med. Assoc..

[71] Kumar N., Goel N. (2023). Recent development of imidazole derivatives as potential anticancer agents. Phys. Sci. Rev..

[72] Information., National Center for Biotechnology Information PubChem Compound Summary for CID 5419, Tetrahydrozoline. https://pubchem.ncbi.nlm.nih.gov/compound/Tetrahydrozoline.

[73] Information., National Center for Biotechnology Information PubChem Compound Summary for CID 2803, Clonidine. https://pubchem.ncbi.nlm.nih.gov/compound/Clonidine.

[74] Hasson H. Generalized Onset Tonic-Clonic Seizures. https://www.medlink.com/articles/generalized-onset-tonic-clonic-seizures.

[75] Bersani F.S., Corazza O., Simonato P., Mylokosta A., Levari E., Lovaste R., Schifano F. (2013). Drops of madness? Recreational misuse of tropicamide collyrium; early warning alerts from Russia and Italy. Gen. Hosp. Psychiatry.

[76] Bellman V., Ukolova A., Erovichenkova E., Lam S., Srivastava H.K., Bruce J., Burgess D.M. (2022). Abuse of tropicamide eye drops: Review of clinical data. Braz. J. Psychiatry.

[77] Bhatia A., Mohan S., Reddy S. (2022). Falling prey to superglue ocular injuries: A case series. Kerala J. Ophthalmol..

[78] Cole J. (2010). Off-label: Technically unproven, but not out of bounds: Optometrists routinely prescribe myriad drugs off-label--just be sure you are following the proper standard of care. Rev. Optom..

[79] Petkova V., Georgieva D., Dimitrov M., Nikolova I. (2023). Off-Label Prescribing in Pediatric Population—Literature Review for 2012–2022. Pharmaceutics.

[80] Vosgerau J., Simonsohn U., Nelson L.D., Simmons J.P. (2019). 99% impossible: A valid, or falsifiable, internal meta-analysis. J. Exp. Psychol. Gen..

[81] Alghamdi S.S., Suliman R.S., Almutairi K., Kahtani K., Aljatli D. (2021). Imidazole as a Promising Medicinal Scaffold: Current Status and Future Direction. Drug Des. Devel Ther..

